# Mechanical Bed for Investigating Sleep-Inducing Vibration

**DOI:** 10.1155/2017/2364659

**Published:** 2017-07-09

**Authors:** Hitoshi Kimura, Akisue Kuramoto, Yuma Inui, Norio Inou

**Affiliations:** Department of Mechanical and Control Engineering, Tokyo Institute of Technology, Tokyo, Japan

## Abstract

In running cars or trains, passengers often feel sleepy. Our study focuses on this physiological phenomenon. If a machine can reproduce this phenomenon, it is feasible to put a person, such as an insomnia patient or an infant, to sleep without any harmful effects. The results of our previous study suggest that low-frequency vibration induces sleep. This report describes a new mechanical bed for inducing sleep and discusses the effects of different vibration conditions. The new bed has two active DOFs in the vertical and horizontal directions to examine the anisotropy of sensation. The bed includes three main parts: a vertical driver unit, a horizontal driver unit, and a unique 2-DOF counterweight system to reduce driving force and noise. With regard to motion accuracy, the maximum motion error in the vertical direction lifting 75 kg load was only 0.06 mm with a 5.0 mm amplitude of a 0.5 Hz sinusoidal wave. The results of excitation experiments with 10 subjects showed a significant difference in sleep latency between the conditions with vibration and without vibration. Furthermore, the average latency with insensible vibration (amplitude = 2.4 mm) was shorter than that with sensible vibration (amplitude = 7.5 mm). These results suggest the ability of appropriate vibration to induce sleep.

## 1. Introduction

The control of sleeping time is essential to modern society. The development of an effective method to induce sleep without harmful effects will bring remarkable benefits for insomnia patients, babies, and employees that work nonstandard shifts (e.g., long-distance drivers). To realize a sleep-inducing method, this study focuses on the phenomenon wherein passengers often fall asleep in running cars or trains. Conceivable sleep-inducing factors in mechanical environments include light, temperature, sound, vibration, and oxygen density. In our previous study, a tendency is observed where the low-frequency vibration of a running train make people fall asleep [1]. This paper describes a new mechanical bed designed to elucidate the detailed vibration conditions necessary to induce sleep. Various mechanical cradles designed to help babies sleep are commercially available; however, few studies have investigated the relationship between vibration and sleep induction. Although many studies and the ISO have defined uncomfortable vibration [2–4], few studies have discussed comfortable vibration [5]. Kitado et al. suggested that the vibration of running trains with 1/f fluctuation has a sleep-inducing effect [6, 7]. The authors have investigated the relationship between sleeping passengers and the vibration of running trains to identify effective vibration for inducing sleep [8]. In that study, the authors did not find a significant relationship between the number of sleeping passengers in running trains and the 1/f fluctuation of their vibration; however, the results suggested that low-frequency vibration, less than 2.0 Hz, tended to induce sleep. The results also showed that a certain magnitude of jerk (i.e., more than 0.2 m/s^3^) disturbs sleep [9, 10]. Thus, our past study indicated that smooth vibration without jerking is required for a mechanical environment to induce sleep. This conclusion is in agreement with studies showing that large jerks impair the comfortableness of vehicles [11, 12]. To reproduce a sleep-inducing environment with a simple mechanism, the authors examined several subjects with a 1-DOF exciter bed [13]. The results showed that a frequency of 0.5 Hz and no fluctuation were optimal conditions to induce sleep. Altmann reports that vibrations with frequency less than 2.0 Hz have a possibility of resonance at a part of the body depending on the subject's posture; however, the resonance vanishes at about 0.5 Hz [14]. A research of motion sickness reported by O'Hanlon et al. showed that 0.2 Hz vibration caused sickness which recovered at about 0.5 Hz [15]. Of course, there remain unknown factors about the relationship between vibration and feeling; these studies agree that 0.5 Hz vibration is not uncomfortable. However, our bed could not examine the anisotropy of body sensation. Thus, this study aims to investigate the detailed conditions of vibration that effectively induces sleep. This paper describes a new prototype mechanical bed that has two active DOFs in the horizontal and vertical directions. The bed has a unique counterweight system with two passive DOFs to decrease the required motor torque and operating noise. Experiments in which sleep latencies were measured on the basis of brain waves under several excitation conditions were conducted with 10 subjects.

## 2. Mechanism of the New Mechanical Bed

### 2.1. Design of the Overall Structure

**Figure 1:**
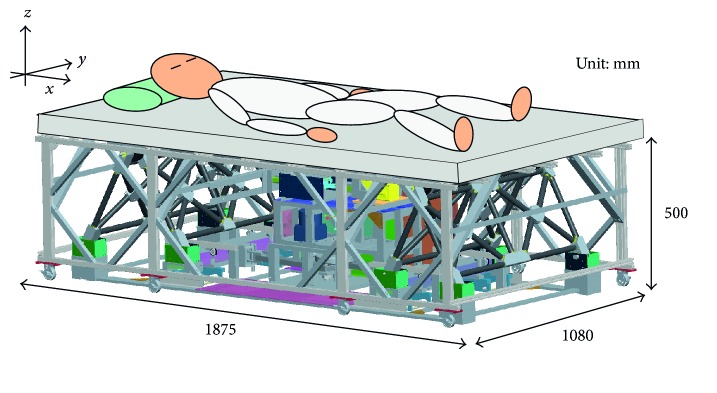
Schematic of the new mechanical bed.

The mechanical bed is designed to stimulate a human body within two directions (horizontal and vertical). Accurate and quiet motion is necessary because this study also aims to determine the vibrational conditions that induce sleep. Since the vertical driving mechanism must lift the subject, the required force would be enormous with a normal mechanism. Although the required force for horizontal motion is lower than that for vertical motion, the movement of the center of mass (hereafter referred to as COM) will cause errors in the vibrational motion and result in large operating noise. To avoid these problems, the authors designed a new counterweight system to generate both vertical and horizontal motion using one weight. This counterweight system decreases the forces required for vertical motion and the COM's movement. The resulting new mechanical bed comprises three main mechanisms: a vertical driving unit, a horizontal driving unit, and a counterweight system.  Figure 1 shows the overall structure of the bed. Hereafter, this paper uses the Cartesian coordinate system shown in Figure 1.

### 2.2. Vertical Driving Unit

**Figure 2:**
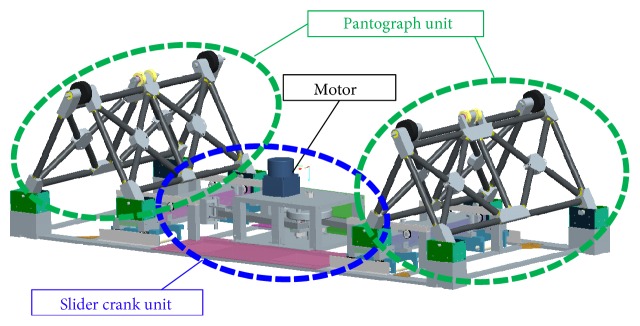
Schematic of the vertical driving unit.

Figure 2 shows the driving mechanism for vertical vibration. The driving mechanism comprises a pair of pantograph mechanisms connected to a slider crank mechanism. The slider crank is driven by the motor, and the pantograph works to elevate the bed frame. The pantograph mechanisms are arranged centrosymmetrically with the motor axis serving as the symmetric center. Within this mechanism, the bed frame is supported by passive wheels at the four corners to achieve stable motion.

**Figure 3:**
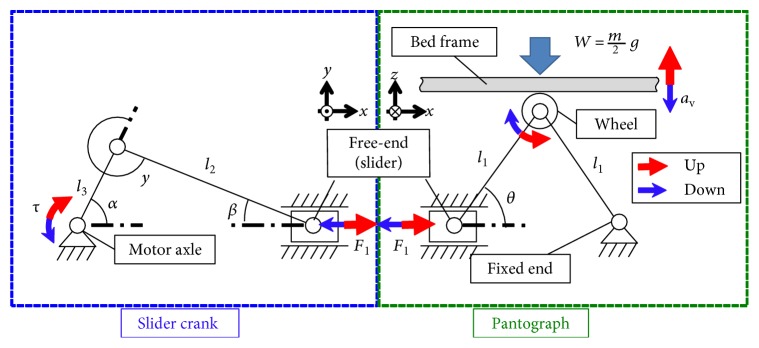
Kinematic diagram of the vertical driving mechanism.

The force required for motion in the vertical direction *F*_v_ is represented by the following equation:

(1) $$\begin{array}{c} F_{v}=\frac{m\left(g+a_{v}\right)}{4\;\tan\;\theta }, \end{array}$$

where *m* is the total mass of vibrational motion including the human subject and bed frame, *g* and *a*_v_ are the accelerations of gravity and in the vertical direction, respectively, and *θ* is the angle of the toggle mechanism linkage shown in Figure 3. The motor torque *τ* can be derived from *F*_v_ and the kinematics as follows:

(2) $$\begin{array}{c} \tau =\frac{2F_{v}l_{3}}{r}\left(\sin\;\alpha +\frac{l_{3}\sin\;2\alpha }{2\sqrt{l_{2}^{2}-l_{3}^{2}\sin^{2}\alpha }}\right), \end{array}$$

where *r* is the reduction ratio of the gearhead of the motor and *l*_*n*_ (*n* = 1, 2, 3) is the link length, as shown in Figure 3. Assuming that the maximum weight of a subject is 100 kg, the maximum required torque is approximately 2.0 Nm. The actual required force is considerably smaller than this maximum torque because the total vertical load, which includes the human body and the bed frame, is supported by a counterweight mechanism, as will be described later. From the viewpoint of safety, this study adopts a stepping motor with sufficient torque to generate 12.0 Nm. Thus, the bed can support the subject without the supporting force of the counterweight mechanism.

### 2.3. Horizontal Driving Unit

**Figure 4:**
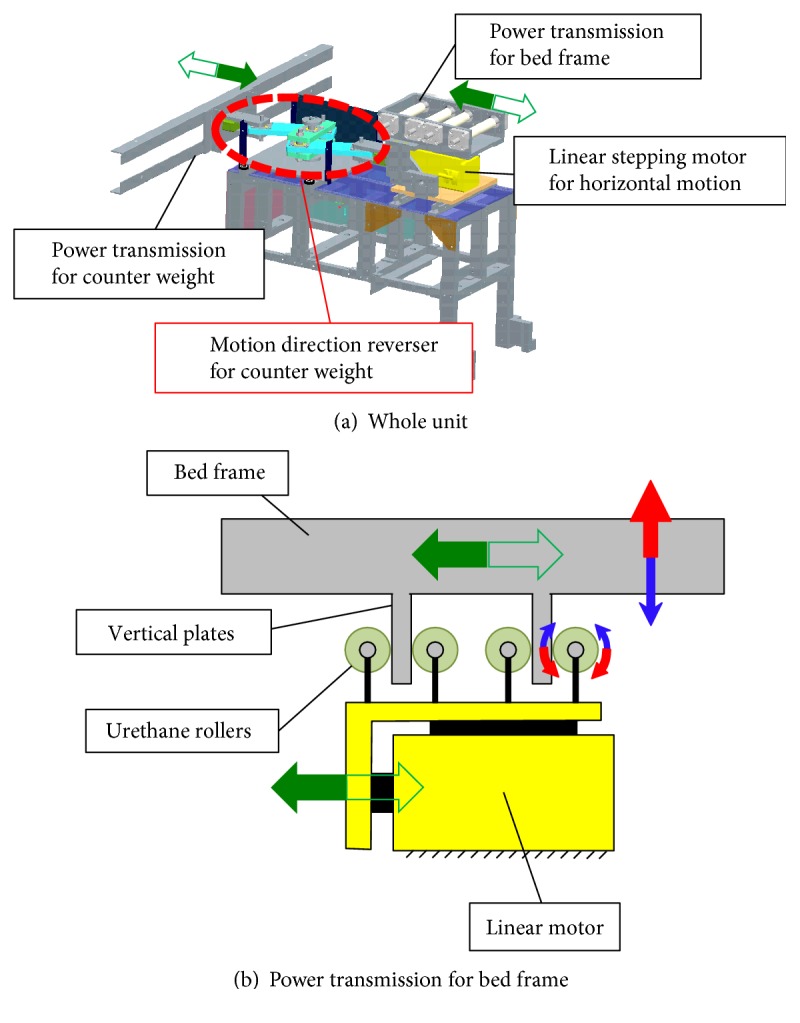
Schematic of the horizontal driving unit.

Figure 4 shows the schematic of the horizontal driving unit. The unit generates symmetrical motion for the bed frame and the counterweight (Figure 4(a)). The symmetrical motion is realized by a direction reverser with a slider crank mechanism. Because the bed frame moves vertically, free vertical motion is required for the horizontal driving unit. As shown in Figure 4(b), two vertical plates are fixed under the bed frame. The plates are grasped by four rollers that are fixed on the linear motor. In this mechanism, the vertical and horizontal motions are independent.

**Figure 5:**
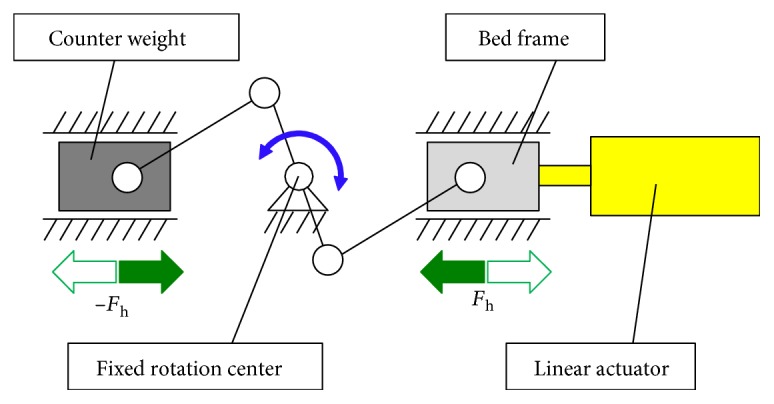
Kinematic diagram of the horizontal driving mechanism.

The bed is equipped with a counter weight for stable and silent motion. The counter weight supports both horizontal and vertical directions. The counter weight moves in the opposite direction of the bed frame as shown in Figure 5. When the bed moves with the horizontal acceleration *a*_h_, considering counterweight motion, the required force *F*_h_ in the horizontal direction is represented by the following equation:

(3) $$\begin{array}{c} F_{v}=2ma_{h}. \end{array}$$

From this equation, the maximum required force of the actuator is approximately 100 N because the maximum acceleration in the horizontal direction is less than 0.5 m/s^2^. Although the counterweight increases the required force, the COM is kept almost constant. This balanced mechanism produces silent motion and suppresses vibration. Moreover, the force required in the horizontal direction is not extremely large. The maximum force of the linear actuator in this bed is approximately 500 N.

### 2.4. Counterweight Unit

**Figure 6:**
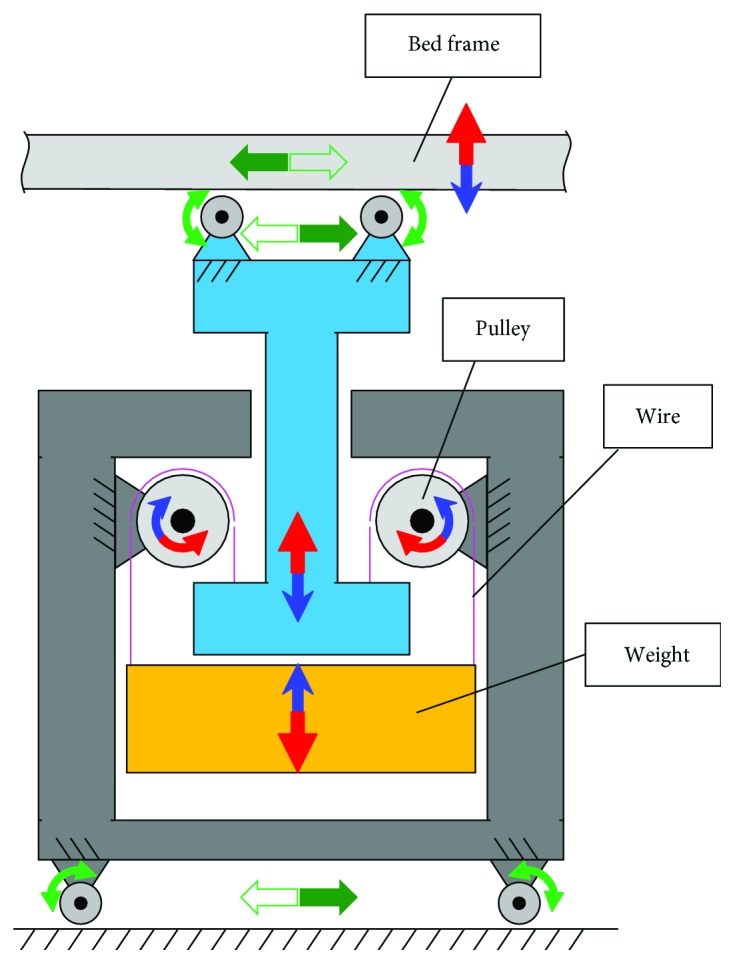
Schematic of counterweight unit.

The schematic of the counterweight unit is shown in Figure 6. The wire and pulley mechanism and the wheel mechanism keep the balance of vertical and horizontal directions, respectively. In the vertical direction, the weight rises (lowers) when the bed frame lowers (rises). With this counterweight mechanism, the counterweight independently moves in both directions and keeps the position of the COM constant. Even if the weight balance is not perfect, the required vertical force is still very small. This mechanism also suppresses the mechanical sound. These considerations are considerably important for providing a comfortable environment for the sleeping experiments.

## 3. Experimental Results and Discussion

### 3.1. Prototype 2-DOF Bed Motion

**Figure 7:**
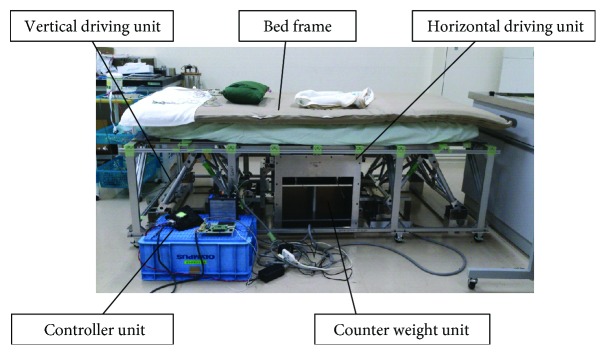
Overview of prototype 2-DOF bed.

**Figure 8:**
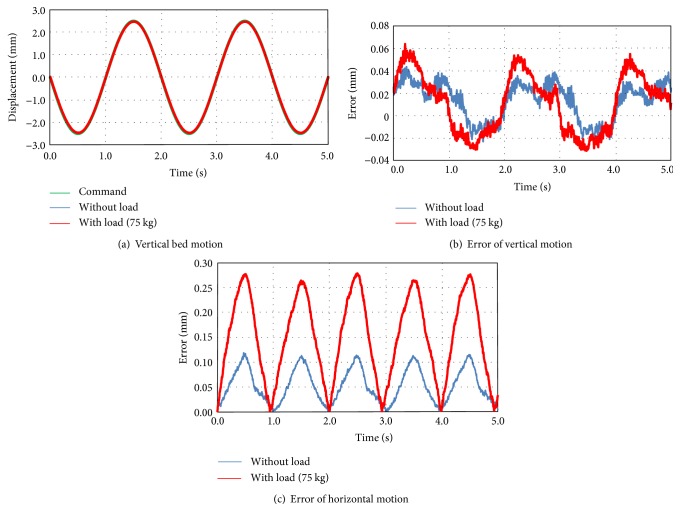
Motion accuracy of the bed (5.0 mm amplitude at 0.5 Hz).

The overall view of the prototype bed is shown in Figure 7. The vibration of this bed is driven by two motors for the vertical and horizontal directions. A microprocessor unit (MPU) independently controls each motor driver, and the frequency and amplitude are variable during the bed excitation. On the basis of our previous research and ISO standards [2, 3], simple vibration of sinusoidal wave with large amplitude tends to cause motion sickness. Although the vibrations of running cars or trains include more than 10 mm amplitude, it is obvious that sleeping is almost impossible under a continuous large amplitude. Authors also confirmed that a sinusoidal vibration with an amplitude of 10 mm or more causes the subject to get motion sickness with another mechanical bed. In addition, a vibration with a frequency of 2.0 Hz or more may not contribute to induce sleep [8]. This tendency is intuitive referring that the natural frequencies of lymph in semicircular canals is about 1.0 to 2.0 Hz [2]. Thus, for each direction, the maximum amplitude and frequency is defined as 10 mm and 2.0 Hz, respectively.  Figure 8(a) shows the difference between the command and actual motion in the vertical direction without load. The actual motion of the bed frame was measured using a KEYENCE laser displacement sensor IA2000. Two sensors are fixed on the ground, and the displacements of the bed frame are measured in both horizontal and vertical directions with high accuracy. The motion accuracy was also confirmed without load and with 75 kg load of human body. The maximum error of the vertical motion is less than 0.06 mm under a sine wave vibration with an amplitude of 5.0 mm at 0.5 Hz (Figure 8(b)). In terms of a machine which can drive the human body, this motion error is extremely small. Meanwhile, the maximum error of the horizontal motion is 0.28 mm with 75 kg human body, which is larger than that of the vertical direction (Figure 8(c)). The horizontal motion error might be caused by urethane rollers for driving force transmitter (Figure 4(b)) which has a certain flexibility for noise reduction. However, even the maximum error in the horizontal direction is less than 0.3 mm and this is enough accuracy for a sleep-inducing experiment. The error of horizontal motion might be reduced by replacing the urethane rollers with stiff parts.

### 3.2. Excitation of Subjects

The 2-DOF mechanical bed is able to provide 2-DOF motion; however, 2-DOF motion includes infinite variation of trajectories. It is not easy to narrow down the candidates of 2-DOF motions without considering the effect of 1-DOF vibration. Based on this reason, this study focuses on the effect of 1-DOF vibration as the first step.

In the first step of the experiment, the excitation frequency was kept at 0.5 Hz because this was determined to be the optimal frequency to induce sleep using the previous 1-DOF exciter bed [13]. Using a cover that wraps around the excitation mechanism, the noise was approximately 43 dB. Although the noise level was very low compared to the environment of daily living (45 dB) [16], all subjects use earplugs because the sound of the motor may be offensive to some subjects.

Ten healthy men aged between 21 and 27 were recruited as subjects in this experiment. The eligibility criteria included (1) no history of sleep disorders and (2) no motion sickness. The experimental protocol adhered to the ethical standards of Tokyo Tech and was authorized by the committee (#A14014). The tested excitation amplitudes were 0, 2.4, 5.0, and 7.5 mm in each direction. Because the amplitude of the previous 1-DOF bed was 2.4 mm, this study also used 2.4 mm instead of 2.5 mm as the amplitude. The subjects were excited in the horizontal or vertical direction to investigate the anisotropy of body sensing, and sleep latency was measured from the subjects' brain waves. The subjects' sensory perceptions of the bed were also collected using a questionnaire.

The brain wave is measured by general electrode made of silver metal with brain-wave amplifier BA1008 manufactured by Digitex Lab. The output voltage is amplified 100,000 times with this system. Sampling rate is 200 Hz and band-pass filter from 1.5 to 30 Hz is applied to the signal. The frequency band is defined for measurement of brainwaves *α* (8–14 Hz), *β* (14–38 Hz), *δ* (0.5–4 Hz), and *θ* (4–8 Hz). These waves are generally used as the indicator of the stages including REM. Low-frequency waves are not very important in our study because the brain waves with low frequency such as *δ* and *θ* are observed on the deep sleep stages 3 and 4. This study focuses on the sleep latency and does not deal with such deep stages. As is well known in the art, hump and K-complex are characteristic waveforms indicating the early stage of sleep. This study adopted these waveforms as the indicators of sleep. Rechtschaffen and Kales method [17], a standard of brain wave discrimination, is used to identify the waveforms.

**Figure 9:**
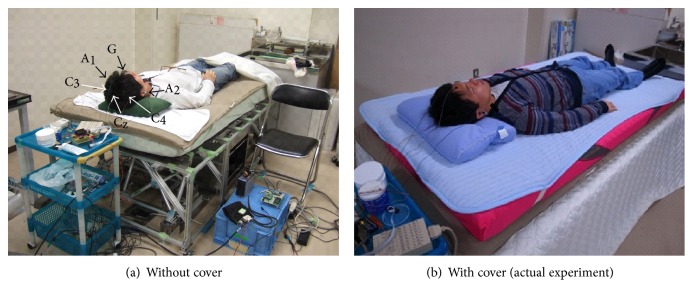
Overview of the excitation experiment.

In order to avoid sleep disturbance, the number of electrodes should be suppressed as little as possible. This study measured the brain waves of Cz, C3, and C4 within the 10–20 method (general EEG method) [18]. The waves of 3-points provide sufficient information to detect hump and K-complex. The voltages of C3 and Cz are refereed by A1 (left earlobe). The voltage of C4 is refereed by A2 (right earlobe). The voltage of G is used to remove common noise. The overview of the experiment and the arrangement of the electrodes are shown in Figure 9. The mechanical part of the bed is covered by a thick cloth to decrease the mechanical sound.

**Figure 10:**
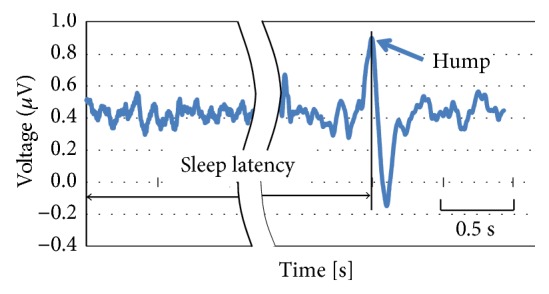
Definition of sleep latency based on brain waves.

Herein, sleep latency is defined as the duration of time between the beginning of excitation and when the hump or K-complex is observed in the brain waves (Figure 10). Of course, the result of sleep latencies indicates different values every time. This study adopted an average value of the latencies, and the number of measurements was at least three times for each subject.

**Figure 11:**
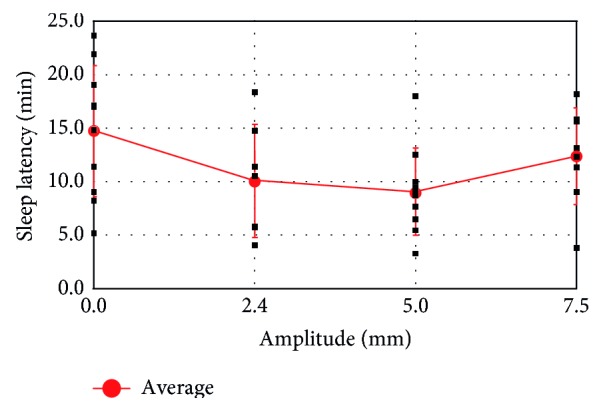
Average sleep latency with 0.5 Hz horizontal excitation.

Average sleep latency was measured under excitation in the horizontal direction, and the results are shown in Figure 11. Without excitation, the average latency was 14.7 min. In contrast, under a 5.0 mm amplitude excitation, the average latency was 9.0 min. At an amplitude of 2.4 mm, the average latency was 10.1 min, although almost all of the subjects could not sense the excitation (based on the responses to the questionnaire). This latency obtained at a 2.4 mm excitation is almost the same as that observed at a 5.0 mm amplitude. In contrast, for excitation at an amplitude of 7.5 mm, the subjects reported that it was easy to feel the vibration. In addition, the corresponding average latency with the amplitude of 7.5 mm was 12.4 min, which is longer than the latencies with the amplitudes of 5.0 mm and 2.4 mm. This result suggests that low-amplitude vibration in the horizontal direction is effective for inducing sleep. This assumption supports the description of human exposure to whole-body vibration in ISO standards [2].

**Figure 12:**
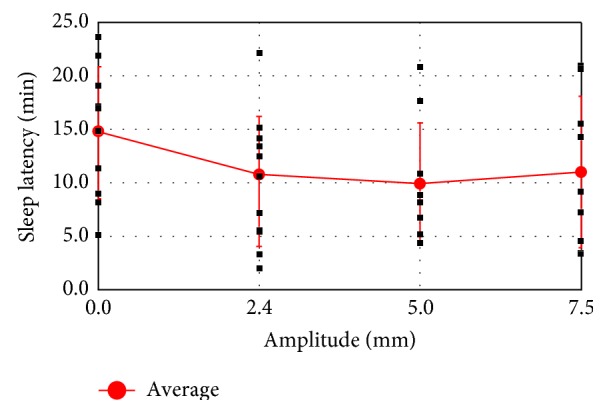
Average sleep latency with 0.5 Hz vertical excitation.

The effect of excitation in the vertical direction was also examined, as shown in Figure 12. The average sleep latencies for vertical excitation at amplitudes of 2.4, 5.0, and 7.5 mm were 10.8, 9.9, and 11.0 min, respectively. The latency for 2.4 mm vertical excitation was also shorter than that for the no-excitation condition (14.7 min). As for horizontal excitation at 2.4 mm, the subjects reported that they could not feel the vertical excitation at 2.4 mm.

The statistical chart of the experimental results is shown in Table 1. The outliers were omitted from the analysis according to the Smirnov-Grubbs test [19]. The most important results are the average sleep latencies with and without excitation, which exhibit a significant difference.

Using statistical methods, this study aimed to detail the ability of excitation to induce sleep. Although the results for horizontal and vertical excitation were slightly different, no statistical significance was found. Furthermore, no significant differences in sleep latency were found among different amplitudes of excitation. The obtained data were then divided into two groups, that is, with and without excitation. These groups were compared using the F-test of equality of variances. The *F*-value was 1.30, and the critical value of the F-distribution was 2.06 under *α* of 0.05 (*α* = significance probability). These values indicate that the two groups have the same variance. The groups were then compared using two-sample *t*-tests assuming equal variance. The calculated *t*-value was 2.28, and the critical value of the *t*-distribution was 2.00 under *α* of 0.05. These results suggest that there is a significant difference between the excitation group and the no-excitation group in terms of average sleep latency. Moreover, the value of Cohen's *d* was 0.79. This value allows us to conclude that appropriate excitation had the effect of inducing sleep, although the statistical power was merely 0.62 due to the small number of subjects. With respect to detailed condition of the effective excitation, we plan to increase the number of subjects in future studies.

As is well known, humans cannot feel his/her displacement but acceleration. A human detects vibration not only the semicircular canals but also each body part such as the pacinian corpuscle. ISO 2631 series [2, 3] describe the standards of whole body vibration. Appendix C of ISO 2631-1 describes that 15 mm/s^2^ is the median of the least acceleration which a human who has exquisite sensitivity can feel [2]. On the basis of this description, 0.5 Hz sinusoidal wave with the amplitude of 5.0 mm generates 25 mm***/***s^2^ (Table 2). This acceleration is on the borderline between sensible and insensible vibrations. Actually, the average sleep latencies of 2.4 mm and 5.0 mm are almost the same. Meanwhile, the acceleration is 37 mm/s^2^ when the amplitude is 7.5 mm. In the worst case, the vibration causes motion sickness. From the results of the sensory test, a large acceleration does not induce sleep but causes an uncomfortable feeling. This tendency is natural even in view of daily life. Our result suggests a possibility that a sleep-inducing phenomenon correlates not only semicircular canals but whole body sensors such as the pacinian corpuscle. In other words, these organs might detect very small accelerations less than 25 mm***/***s^2^ which humans cannot recognize, and affect inducing sleep. This assumption can explain the result.

**Figure 13:**
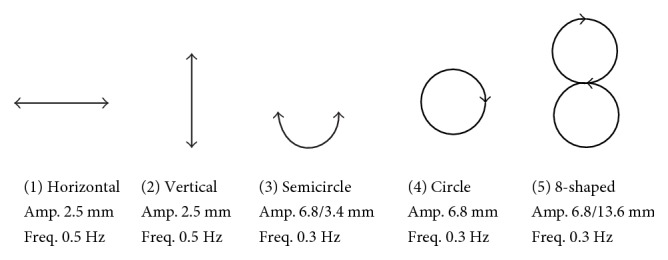
Examined trajectories of 2-DOF excitation.

In order to confirm the bed performance, this study also examined 2-DOF excitation. As a preliminary experiment, 5-trajectories including 1-DOF and 2-DOF motions are examined as shown in Figure 13. The maximum accelerations are the same value of 12 mm/s^2^ for result comparison between the trajectories. The 2-DOF trajectories are realized by calculated speed from differentiated trajectory. The speed of the bed is controlled by position command (the number of steps) at regular intervals because the bed is driven by stepping motors. For example, in the case of a circular motion with a radius *A* and rotation speed *ω*, the trajectory is expressed by position vector **p** = (*A*cos*ωt*, *A*sin*ωt*). However, it is impossible to realize the speed of (*d ***p**_*x*_/*dt*, *d ***p**_*y*_/*dt*) = (−*Aω*sin*ωt*, *Aω*cos*ωt*) from the static state ((*v*_*x*_, *v*_*y*_) = (0, 0)) directly. Coordinated accelerations in horizontal and vertical (*x* and *y*) directions are necessary to realize the 2-DOF motion. At first, the velocity in the vertical direction is accelerated from 0 to *Aω*cos*ωt*. Meanwhile, the velocity in the horizontal direction is kept 0. When the vertical speed reaches *Aω*cos*ωt* (*t =* 0), the horizontal motion is started with the velocity of −*Aω*sin*ωt* (=0 when *t* = 0). With this method, a circular bed motion can be generated. Other 2-DOF motions are realized in the similar ways. Of course, by differentiation of the trajectory directly, obtaining the speed command is also possible. With 3 subjects, there was no clear difference of sleep latencies and questionnaire results between 1-DOF and 2-DOF motions. Increasing the number of subjects and statistical analysis are our future works.

## 4. Conclusion

We developed a new, active 2-DOF mechanical bed that excites the human body to induce sleep. In the prototype bed, a counterweight system decreases the required motor torque and driving noise, allowing it to achieve silent and smooth excitation. The vertical and horizontal excitation of the bed can be controlled independently. In both the horizontal and vertical directions, the motion error is almost only 0.02 mm for a sine wave with a frequency of 0.5 Hz and an amplitude of 5.0 mm. The brain waves of human subjects were measured both with and without excitation. Several amplitudes were examined by sinusoidal excitation at a constant frequency of 0.5 Hz in both the horizontal and vertical directions. Although an optimal amplitude of excitation was not identified, we observed an evident statistical difference in average sleep latency between the with- and without-excitation groups. Particularly, very low-amplitude vibrations also induce sleep although subjects cannot recognize the vibration. These results indicate that an effective vibration can induce sleep.

## Figures and Tables

**Table 1:** Statistical results of sleep latencies using the Smirnov-Grubbs test.

	No exc.	Horizontal excitation			Vertical excitation		
Amplitude	0	2.4	5.0	7.5	2.4	5.0	7.5
Ave. latency [min]	14.7	10.1	9.0	12.4	10.8	9.9	11.0
Min. latency [min]	5.1	4.0	3.2	9.0	2.0	4.4	3.5
Max. latency [min]	21.9	18.3 (29.7)	18.0 (26.6)	15.6	15.2	17.6	21.0
Std. deviation	6.1	5.3	4.1	4.5	6.1	5.7	7.1
Variance	34.0	23.8	14.9	17.8	33.4	28.6	44.2
Conf. interval [%]	4.2	4.5	2.8	3.5	3.4	3.5	4.3
Sample number	10	7	10	8	11	9	9
Number of exclusion	2	3	2	3	1	2	1
Outliers in exclusion	0	1	1	0	0	0	0
Number of experiments	12	10	12	11	12	11	10

Values in parentheses are omitted as outliers.

**Table 2:** Acceleration values for each amplitude under 0.5 Hz sine wave.

Amplitude (mm)	2.4	5.0	7.5
Acceleration (mm/s^2^)	12	25	37
